# Co-ordination in Morphological Leaf Traits of Early Diverging Angiosperms Is Maintained Following Exposure to Experimental Palaeo-atmospheric Conditions of Sub-ambient O_2_ and Elevated CO_2_

**DOI:** 10.3389/fpls.2016.01368

**Published:** 2016-09-15

**Authors:** Christiana Evans-Fitz.Gerald, Amanda S. Porter, Charilaos Yiotis, Caroline Elliott-Kingston, Jennifer C. McElwain

**Affiliations:** ^1^Earth Institute, O’Brien Centre for Science, University College DublinDublin, Ireland; ^2^School of Biology and Environmental Science, University College DublinDublin, Ireland; ^3^School of Agriculture and Food Science, University College DublinDublin, Ireland

**Keywords:** vein density, stomatal density, co-ordination, plant growth chambers, palaeo-atmosphere

## Abstract

In order to be successful in a given environment a plant should invest in a vein network and stomatal distribution that ensures balance between both water supply and demand. Vein density (*D*_v_) and stomatal density (SD) have been shown to be strongly positively correlated in response to a range of environmental variables in more recently evolved plant species, but the extent of this relationship has not been confirmed in earlier diverging plant lineages. In order to examine the effect of a changing atmosphere on the relationship between *D*_v_ and SD, five early-diverging plant species representing two different reproductive plant grades were grown for 7 months in a palaeo-treatment comprising an O_2_:CO_2_ ratio that has occurred multiple times throughout plant evolutionary history. Results show a range of species-specific *D*_v_ and SD responses to the palaeo-treatment, however, we show that the strong relationship between *D*_v_ and SD under modern ambient atmospheric composition is maintained following exposure to the palaeo-treatment. This suggests strong inter-specific co-ordination between vein and stomatal traits for our study species even under relatively extreme environmental change. This co-ordination supports existing plant function proxies that use the distance between vein endings and stomata (*D*_m_) to infer plant palaeo-physiology.

## Introduction

Global diversity in plant and leaf architecture reflects a plasticity in morphology that allows plants to survive in a range of environments (Díaz et al., 2016). In this current era of rapid climate change, understanding the relationships between plant morphological traits and how they might be influenced by the surrounding environment is of the utmost importance, enabling predictions of plant responses over the coming decades as atmospheric carbon dioxide (CO_2_) rises. Plants are a critical component of the hydrological cycle, influencing the amount of water vapor that is returned to the atmosphere via the process of transpiration (Rodell et al., 2015). The predicted future increases in CO_2_ and global temperatures will have an impact on plant physiological function and morphological traits and will consequently influence the hydrological cycle (Gedney et al., 2006; Betts et al., 2007). The present study focuses on vein and stomatal density (SD), two plant morphological traits that play a pivotal role in the transpirational pathway, and attempts to understand how one may influence the other as a plant encounters environmental change.

Stomata are microscopic pores on a leaf surface that regulate gas exchange. CO_2_ from the atmosphere which is essential for photosynthesis is exchanged for water vapor from the inside of the leaf (Jones, 1992). Stomata respond to environmental cues, opening in response to increasing light, low carbon dioxide, and high humidity (Assmann, 1999; Outlaw, 2003; See review by Roelfsema and Hedrich, 2005; Vavasseur and Raghavendra, 2005; Shimazaki et al., 2007; Lawson, 2009). Stomatal opening results in an increase in stomatal pore aperture which leads to an increase in both carbon uptake and water loss from the leaf. SD is the number of stomata per mm^2^ of leaf tissue and it is determined by various genetic (Nadeau and Sack, 2002; Shpak et al., 2005) and environmental factors (McElwain and Chaloner, 1995; Woodward and Kelly, 1995; Casson and Gray, 2008). A change in SD alters gas exchange along the plants’ diffusional pathway, influencing transpiration and therefore water demand. Veins are found in the leaves of plants, and are differentiations of the vascular bundles that transport water and nutrients from the soil to leaves, as well as sucrose from leaves to the storage sites of the plant (Sack and Holbrook, 2006). A network of major and minor veins (some species only have major veins) carries water throughout the leaf tissue to the stomata where it is lost to the atmosphere as water vapor. Vein density (*D*_v_) is the length of veins per leaf area (mm mm^-2^), and in angiosperms it is determined predominantly by the minor veins, as they make up >80% of the total vein length of the leaf (Sack et al., 2012). Minor vein density has been shown to be an important functional plant trait, exerting a strong influence over xylem conductivity (*K*_x_) and outside xylem conductivity (*K*_ox_), parameters that determine leaf hydraulic conductance (*K*_leaf_) (Sack and Frole, 2006; McKown et al., 2010). Thus it could be said that in the same way that SD and size influence the water demands of a plant, the vein architecture influences its water supply.

In order to be successful in a given environment a plant should invest in a vein network and stomatal distribution that ensures balance between both water supply and demand. Maintaining this balance via co-ordinated shifts in venation and stomatal traits should ensure that the plant is operating at optimal efficiency in terms of carbon uptake and water loss, conforming to the optimality principle (Sack and Scoffoni, 2013). Previous studies have found a strong relationship between *D*_v_ and SD in response to light (Brodribb and Jordan, 2011) and vapor pressure deficit (Carins Murphy et al., 2014) in certain derived plant species and between SD and transpiration (*T*) across a range of ferns, conifers, and angiosperms from both tropical and temperate ecosystems (Boyce et al., 2009). *D*_v_ and SD have been shown to be strongly correlated with modeled maximum theoretical stomatal conductance (*g*_max_) in a diverse range of Proteaceae species (Brodribb et al., 2013). Furthermore, a recent study using a range of modern and basal plant species grown in greenhouse conditions has also reported a strong correlation between *D*_v_ and *g*_max_ (McElwain et al., 2016b), suggesting that this balance does indeed exist. Moreover, other studies combine anatomical and physiological measurements to uncover the links between the architectural properties of a leaf and photosynthetic potential. For example, a proxy for leaf photosynthetic capacity has been developed based on the mesophyll path length (*D*_m_) between vein endings and stomata. In multi-veined species, veins should be optimally placed to minimize *D*_m_ ensuring maximum photosynthetic capacity (Brodribb et al., 2007). These results together demonstrate the potential link between leaf hydraulic morphology and photosynthetic physiology and also highlight the ability of plants to maintain a balance between leaf phenotypic traits under environmental change.

The co-ordination between water supply (*D*_v_) and demand (SD) traits is critical to plant success and the relationship between the two seems to be conserved across the major plant groups under present day atmospheric conditions (Boyce et al., 2009; Brodribb and Jordan, 2011; Brodribb et al., 2013; Carins Murphy et al., 2014; McElwain et al., 2016b). However, it is not known whether this relationship is maintained when levels of oxygen (O_2_) and CO_2_ in the atmosphere change. Co-ordination between these two morphological traits may have been critical throughout the past 400 million years during times of fluctuating atmospheric O_2_ and CO_2_. Maintaining this balance between water supply and demand may have allowed certain species to operate more efficiently in their environment. For example, it has been widely proposed that the co-evolution of leaf traits (an increase in *D*_v_ and SD) during the Cretaceous decline in atmospheric CO_2_ allowed angiosperms to outcompete other plant groups as they transitioned from predominantly moist to drier habitats (Boyce and Zwieniecki, 2012; de Boer et al., 2012; McElwain et al., 2016b).

Stomatal density has been shown to be inversely proportional to atmospheric CO_2_ (McElwain and Chaloner, 1995; Woodward and Kelly, 1995; Beerling and Woodward, 1996; Royer, 2001; Konrad et al., 2008; Franks and Beerling, 2009a,b) and it has been accepted as a palaeo-environmental proxy for CO_2_ on this basis. Studies examining SD responses to concurrent changes in atmospheric O_2_ and CO_2_ are scarce, as are those investigating *D*_v_ responses to atmospheric change. In one of the few studies, examining both living and herbarium material of *Acer monspessulanum* L. and *Quercus petraea* Liebl, no change was observed in *D*_v_ in response to an increase in CO_2_ from 280 to 350 ppm (Uhl and Mosbrugger, 1999). However, in other studies *D*_v_ has been shown to respond to environmental change (Brodribb and Jordan, 2011; Sack and Scoffoni, 2013; Carins Murphy et al., 2014) and has also been used in models to predict both atmospheric carbon dioxide partial pressure and temperature (Blonder and Enquist, 2014). Furthermore, *D*_v_ has the potential to be a useful palaeo-environmental proxy, as venation networks are often preserved in fossilized plant material. For example, studies have shown an increase in *D*_v_ in angiosperms during the Cretaceous period when CO_2_ was declining (Feild et al., 2011a; Boyce and Zwieniecki, 2012).

**FIGURE 1:**
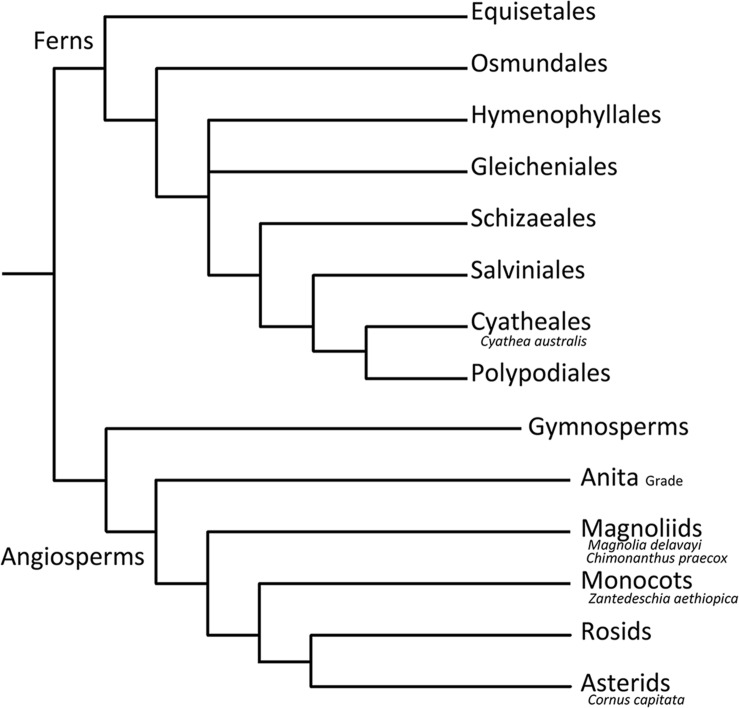
**Diagram illustrating the phylogenetic placement of selected experimental plant species.** Constructed using http://www.mobot.org/MOBOT/research/APweb/.

Using a range of early diverging plant species (**Figure 1**), this study examines for the first time the effect of changing atmospheric conditions on the relationship between *D*_v_, SD, and *g*_max_. Using four angiosperm and one fern species, the plasticity and co-ordination of these morphological plant traits was assessed in a low O_2_/high CO_2_ atmosphere. In the context of this study, co-ordination in plant traits refers to either inter-specific or intra-specific co-ordination. Inter-specific co-ordination is taken to mean an observable trend in morphological plant traits across the experimental species. Intra-specific co-ordination on the other hand, is when the direction of change of plant traits is the same within a single species. We acknowledge that the number of species studied here is relatively small and experimental conditions limited to one palaeo-treatment, and as such, results discussed here are merely intended to be a suggestion of the possible behavior of early diverging plant species under changing atmospheric conditions. Further studies using a wider range of species and palaeo-treatments will build on the current study and will allow more robust conclusions to be drawn.

Results were used to determine the robustness of plant function proxies that rely on co-ordination in morphological traits (such as the use of *D*_m_ as a proxy for palaeo-assimilation rate (Brodribb et al., 2007; Wilson et al., 2015)), specifically when applied at times of the geological past when the atmospheric composition was different from that of today. Theoretical maximum stomatal conductance (*g*_max_) is a plant functional trait calculated using both SD and anatomical measurements of stomatal geometry (Franks and Beerling, 2009a). This trait has been used in palaeo-CO_2_ proxy models (Franks et al., 2014) to infer past CO_2_ levels from the stomatal conductance (*g*_s_) of ancient fossil species. The extent of the relationship between *g*_max_ and operational stomatal conductance (*g*_op_) has been shown in two angiosperm species (Franks et al., 2009; Dow et al., 2014) and more recently in a range of basal angiosperm, gymnosperm, and fern species (McElwain et al., 2016b). Across this range of basal species *g*_max_ and *g*_op_ were found to be strongly related (*r*^2^ = 0.54), with a scaling relationship of *g*_op_ = 0.25 *g*_max_. Therefore *g*_max_ was used in the current study as a means to infer changes in physiological behavior with a change in the concentration of O_2_ and CO_2_ and to relate this to changes in *D*_v_.

## Materials and Methods

### Species and Growth Conditions

Plant species from two different evolutionary plant groups, ferns (*Cyathea australis*) and angiosperms (*Chimonanthus praecox*, *Magnolia delavayi*, *Cornus capitata*, and *Zantedeschia aethiopica*), were grown for approximately 7 months in Conviron (Winnipeg, MB, Canada) BDW-40 walk-in plant growth chambers at PÉAC (Programme for Experimental Atmospheres and Climate), Rosemount Environmental Research Station, University College Dublin. For each of these plant groups, the earliest diverging species obtainable from each plant family was used in order to follow the nearest living relative (NLR) protocol (Mosbrugger, 2009), whereby the responses of extant plant species can be said to reflect the responses of their extinct relatives. Growth conditions were set to ambient (two chambers at 21% O_2_ and 400 ppm CO_2,_ O_2_:CO_2_ ratio of 525) and a palaeo-treatment of low O_2_/high CO_2_ (three chambers at 16% O_2_ and 1900 ppm CO_2,_ O_2_:CO_2_ ratio of 84.21). These conditions represent prehistoric modeled atmospheres (Bergman et al., 2004; Berner, 2009) that likely occurred multiple times throughout the last 400 million years, for example in the Devonian (∼ 419–359 mya), the late Triassic (∼ 218–201 mya), and Jurassic periods (∼ 201–145 mya) (Willis and McElwain, 2014). Plants were given water and nutrients according to the individual species requirements (See Supplementary Table S1). Chambers were set to a 16 h day/8 h night schedule, with day/night temperatures of 20°C/15°C, relative humidity of 65% and a photosynthetic photon flux density (PPFD) of 600 μmol m^-2^ s^-1^.

### SD, *D*_v_, and *g*_max_ Quantification

SD and *D*_v_ were quantified on three leaves per plant, and three to four plants per species per treatment using a modified vein density protocol (Berlyn and Miksche, 1976; Perez-Harguindequy et al., 2013). Leaves were cleared in 5% NaOH, bleached, and then brought through a series of 30, 50, 70, and 100% ethanol. Leaves were then stained using Safranin and Fast Green before being brought back through the ethanol series in the reverse order (100–30% ethanol). Leaves were then suspended in distilled water before being mounted on glass slides for microscopy. For SD quantification, multiple images (on average six images) were taken over an area of approximately 1 cm^2^ per leaf and stomatal counts performed using Image J software inside a superimposed grid of 0.09 mm^2^ on each image (this area was already determined to be the most representative of the entire leaf using the protocol of Poole and Kürschner (1999) from Jones and Rowe (1999)). For *D*_v_ quantification, images were taken on three areas of each leaf (an area of approximately 2 mm^2^ near the tip, center, and bottom of the leaf near the petiole) and using Image J software the length of veins in these areas was manually traced (using a Wacom Intuos4 pen tablet) and *D*_v_ calculated in Excel. Minor *D*_v_ is independent of leaf size and accounts for the majority (>80%) of total vein length per area in most angiosperms (Sack et al., 2012), therefore vein length per area was calculated only on minor veins (quaternary orders upward), major veins being excluded from analysis. Images were taken using a Leica DM2500 microscope with Leica DFC300FX camera (Leica^®^ Microsystems, Wetzlar, Germany) attached and Syncroscopy Automontage (Synchroscopy, Cambridge, UK) digital imaging software was used to impose grids and scale bars on each image.

For *g*_max_ quantification, anatomical measurements of 90 to 120 stomata per species and per treatment were obtained using the same images used for SD determination (See **Table 1** for parameter values). *g*_max_ was calculated using the following diffusion equation (Parlange and Waggoner, 1970; Franks and Beerling, 2009a):

**Table 1:** Measured parameters (± standard deviation) for experimental species.

	SD mm^-2^		*D*_v_ mm mm^-2^		Pore length μm		Pore depth μm		*g*_max_ mmol m^-2^s^-1^	
	Ambient (*n* = 12)	Palaeo (*n* = 12)	Ambient (*n* = 12)	Palaeo (*n* = 12)	Ambient (*n* = 12)	Palaeo (*n* = 12)	Ambient (*n* = 12)	Palaeo (*n* = 12)	Ambient (*n* = 12)	Palaeo (*n* = 12)
*Chimonanthus praecox*	513 ± 103.2	390 ± 102.2	8.3 ± 0.8	7.0 ± 1.0	10.4 ± 1.0	10.9 ± 1.0	3.8 ± 0.3	3.4 ± 0.3	2300 ± 279.0	1923 ± 298.9
*Magnolia delavayi*	343 ± 48.2	316 ± 72.7	6.6 ± 0.5	6.7 ± 1.1	8.9 ± 0.9	8.7 ± 0.8	5.8 ± 0.9	5.6 ± 0.9	1018 ± 210.8	908 ± 201.3
*Cornus capitata*	193 ± 21.8	210 ± 34.6	4.4 ± 0.3	5.1 ± 0.5	10.3 ± 0.5	10.3 ± 0.5	6.5 ± 0.6	5.9 ± 0.6	671 ± 74.3	774 ± 156.5
*Zantedeschia aethiopica*	104 ± 20.4	68 ± 14.7	4.3 ± 0.5	3.8 ± 0.2	15.9 ± 1.7	16.0 ± 0.9	8.6 ± 0.5	8.3 ± 0.8	599 ± 108.6	406 ± 75.0
*Cyathea australis*	171 ± 50.3	145 ± 50.5	3.3 ± 0.2	2.8 ± 0.2	12.4 ± 1.4	13.6 ± 1.7	9.4 ± 0.8	9.4 ± 0.5	649 ± 213.4	635 ± 216.8

*SD, stomatal density, *D*_v,_ vein density, *g*_max,_ maximum theoretical stomatal conductance. *n* = 12 for all species except *Zantedeschia aethiopica* where *n* = 9.*

$$g_{\max}\,=\frac{\left(\frac{dw}{v}\cdot SD\cdot pa_{\max}\right)}{pd+\frac{\Pi }{2}\cdot \sqrt{\frac{pa_{\max}}{\Pi }}}\,\;\;\;\;\;\;\;\;\;\;\;\;\;\;\;\;\;\;\;\;\;\;\;\;\;\;\;\;(1)$$

dw = diffusivity of water vapor at 25°C (0.0000249 m^2^ s^-1^), ν = molar volume of air (0.0224 m^3^ mol^-1^), SD = Stomatal density (m^-2^), *pa*_max_ = max stomatal pore area (m^2^), *pd* = stomatal pore depth (m). The maximum stomatal pore area was calculated (treating the pore as an ellipse) by using stomatal pore length as the long axis, pore length/2 as the short axis and taking the stomatal pore depth as being equal to the width of a fully turgid guard cell (Franks and Beerling, 2009a,b). It is important to note here that guard cells examined in the current study were not experimentally maintained at maximum turgor before anatomical measurements were made. Even though this might lead to a slight underestimation of *g*_max_, this approach is in line with that used in many other palaeo-studies (Franks and Beerling, 2009a,b).

### Vein density responses of a selection of non-angiosperm species from 2009 palaeo-experiment

In order to assess whether other non-angiosperm species show a change in *D*_v_ under different atmospheric compositions, dried plant material from a previous palaeo-experiment was analyzed for *D*_v_. Representatives of both the gymnosperms (*Agathis australis*, *Lepidozamia peroffskyana*, and *Ginkgo biloba*) and ferns (*Osmunda regalis*) were grown for 18 months in walk-in Conviron growth chambers in both an ambient and low O_2_/high CO_2_ atmosphere (Ambient treatment: 20.9% O_2_ and 380 ppm CO_2,_ low O_2_/high CO_2_ treatment: 13% O_2_ and 1500 ppm CO_2,_ O_2_:CO_2_ ratios 552.63 and 86.67, respectively). These species have either parallel or dichotomously branching major veins, therefore it was not necessary to clear and stain leaves for *D*_v_ observation. *D*_v_ was calculated on one leaf per plant and three plants per species per treatment. Leaves were imaged using a Nikon SMZ1000 stereomicroscope with Leica DFC490 camera attached (Leica^®^ Microsystems, Wetzlar, Germany), and using Image J software the length of veins was manually traced and *D*_v_ calculated in Excel in an area between 20 mm^2^ and 60 mm^2^.

### Statistical Analysis

Data were first checked for normal distribution and Generalized Linear Models were run in Minitab (version 16.1.1) statistical software to investigate differences in SD, *D*_v_, and *g*_max_ between treatments. Minitab (version 16.1.1) statistical software was also used for correlation tests, boxplot representation of data, and to graphically display percent changes in SD, *D*_v_, and *g*_max_. RStudio (version 0.99.489) was used for Standardised Major Axis (SMA) regression analysis and for scatterplot representation of data.

## Results

**FIGURE 2:**
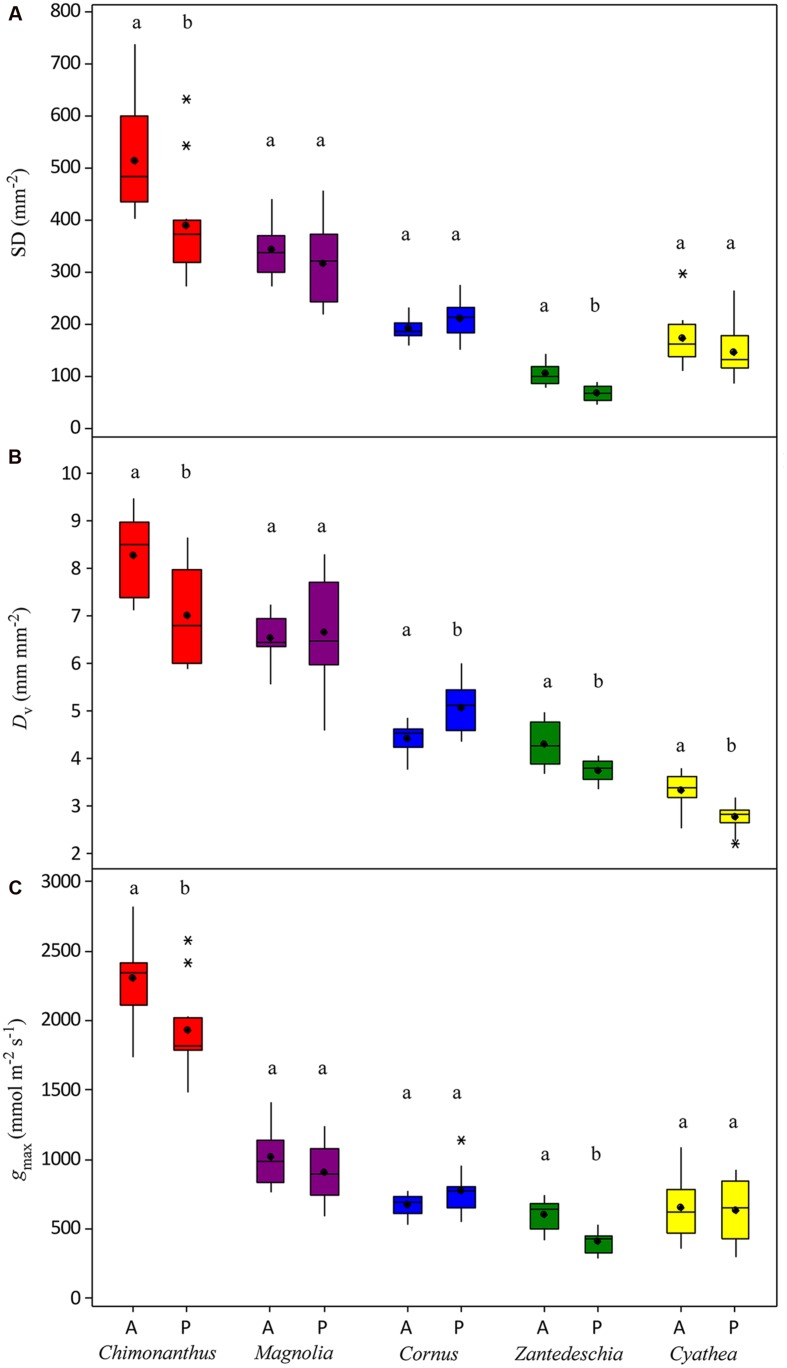
**Changes in (A) SD, (B) *D*_v_, and (C) *g*_max_ of experimental species in response to the palaeo-treatment**. Different letters signify a significant difference between treatments. A, ambient treatment; P, palaeo-treatment. ^∗^symbols denote outliers.

**FIGURE 3:**
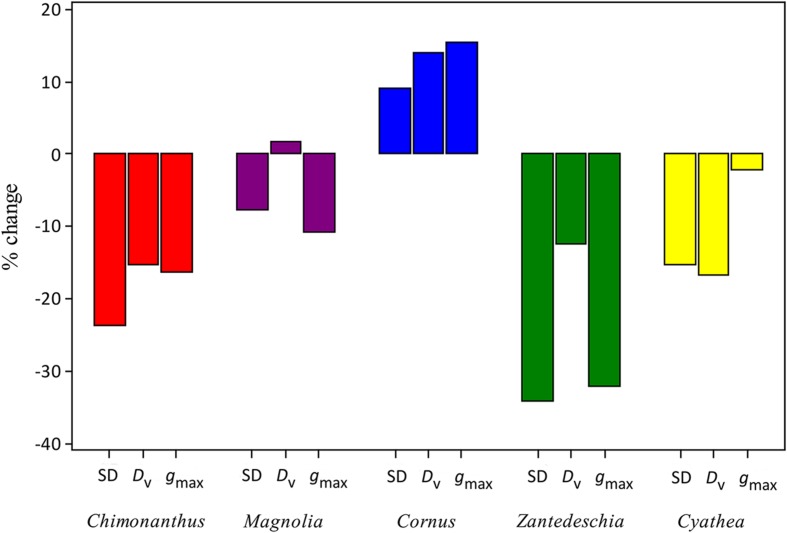
**Percent change in SD, *D*_v_, and *g*_max_ of experimental species**.

Species show a varied and species-specific response in SD, *D*_v_, and *g*_max_ to the palaeo-treatment (**Figure 2**; **Table 1**). A significant decrease in SD is seen in two species (*Chimonanthus praecox* shows a 24% and *Zantedeschia aethiopica* a 34% decrease), *Magnolia delavayi* and *Cyathea australis* show a non-significant yet noticeable decrease (8 and 15%, respectively), and another angiosperm (*Cornus capitata*) shows a small (9%), but non-significant increase (**Figures 2A** and **3**). *D*_v_ shows a similar mix of responses, three of the species show a significant decrease (*Chimonanthus praecox*, *Zantedeschia aethiopica*, and *Cyathea australis* decrease by 15, 12, and 17%, respectively), *Magnolia delavayi* shows a very slight (2%) yet non-significant increase, and *Cornus capitata* shows a significant (14%) increase (**Figures 2B** and **3**). Two of the angiosperm species (*Chimonanthus praecox* and *Zantedeschia aethiopica*) show a significant decrease in *g*_max_ (16 and 32%, respectively) in response to the palaeo-treatment, with *Magnolia delavayi* showing a non-significant decrease (11%), *Cornus capitata* a non-significant increase (15%), and *Cyathea australis* a non-significant decrease (2%) (**Figures 2C** and **3**). See Supplementary Table S2 for results of generalized linear models (*F* and associated *p*-values).

Three out of four angiosperm species (*Chimonanthus praecox*, *Cornus capitata*, and *Zantedeschia aethiopica*) and the fern species *Cyathea australis* demonstrate intra-specific co-ordination of *D*_v_, SD, and *g*_max_ in response to the palaeo-treatment (**Figure 3**). This co-ordination is evident even though one angiosperm species (*Cornus capitata*) shows an increase in all three parameters while the remaining species show a decrease. *Magnolia delavayi* shows co-ordination in two plant traits in response to the palaeo-treatment (SD and *g*_max_), however, co-ordination is lacking between both of these parameters and *D*_v_.

**FIGURE 4:**
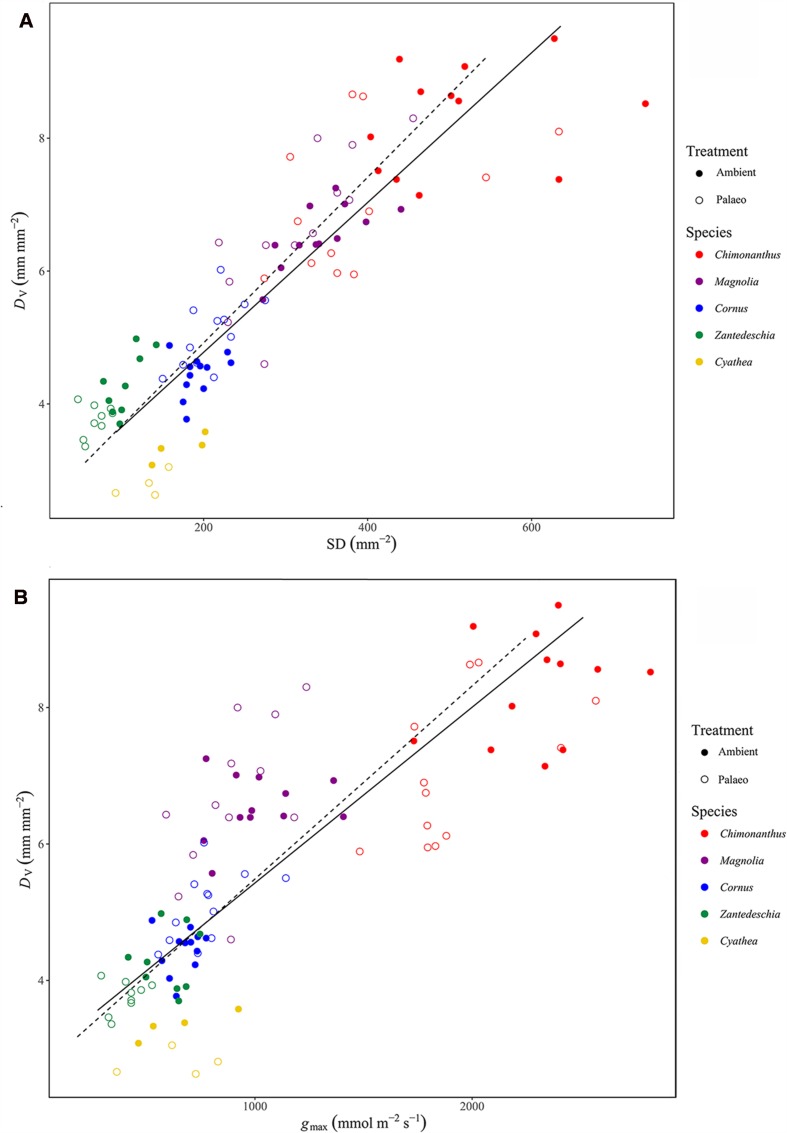
**SMA regression showing the relationship between (A) *D*_v_ and SD, *D*_v_ = 0.01SD + 2.53 for ambient and *D*_v_ = 0.01SD + 2.39 for palaeo-treatment, and (B) *D*_v_ and theoretical maximum stomatal conductance (*g*_max_), *D*_v_ = 0.003*g*_max_ + 2.85 for ambient and *D*_v_ = 0.003*g*_max_ + 2.66 for palaeo-treatment**. Each data point represents a single leaf with the exception of *Cyathea* where data points represent the average per plant.

The positive relationship between *D*_v_ and SD (**Figure 4A**) is strong under ambient conditions (Pearson’s correlation coefficient: *r* = 0.91, SMA regression: *r*^2^ = 0.82) and it persists in the palaeo-treatment (*r* = 0.86, *r*^2^ = 0.73). Similarly, *D*_v_ and *g*_max_ (**Figure 4B**) show a strong relationship under both the ambient (*r* = 0.87, *r*^2^ = 0.76) and palaeo-treatment (*r* = 0.73, *r*^2^ = 0.53). The slopes of the regression lines between *D*_v_ and SD and *D*_v_ and *g*_max_ are not significantly different in the ambient and palaeo-treatments (*D*_v_ = 0.01SD + 2.53 for ambient and *D*_v_ = 0.01SD + 2.39 for palaeo-treatment; *D*_v_ = 0.003*g*_max_ + 2.85 for ambient and *D*_v_ = 0.003*g*_max_ + 2.66 for palaeo-treatment.

## Discussion

### SD, *D*_v_, and *g*_max_ Responses to Low O_2_/High CO_2_

Results of the current study reflect variability in SD responses to atmospheric change. The inverse relationship between SD and CO_2_ has been well documented in the literature using both fossil, herbarium, and living plant material (Woodward and Kelly, 1995; McElwain and Chaloner, 1995; Beerling and Woodward, 1996; Royer, 2001; Konrad et al., 2008; Franks and Beerling, 2009a,b). However, this inverse relationship is not universal across all species (Beerling and Kelly, 1997; Haworth et al., 2013). Stomatal responses to O_2_ are not well documented in the literature to date. The few existing studies, however, show an increase in stomatal index (ratio of stomata to epidermal cells or SI) in response to growth in 35% O_2_ (Beerling et al., 1998), and a range of SD and SI responses to growth in a combined low O_2_/high CO_2_ treatment, as well as to separate low O_2_ and high CO_2_ treatments (Haworth et al., 2013).

The observed decrease in *D*_v_ in the majority of species exposed to the palaeo-treatment is likely a consequence of an overall lower water demand due to stomatal optimisation in a high CO_2_ atmosphere. Reduced *g*_s_ in response to high CO_2_ has been shown in previous studies (Haworth et al., 2013). This overall reduction in SD and *D*_v_ reflects a balance between water supply and demand in the palaeo-treatment, and the overall result would likely be a reduction in allocation of resources to non-essential veins and stomata, and a maximization of resource use. It is important to acknowledge that the SD and *D*_v_ responses observed in the current study cannot be attributed to either low O_2_ or high CO_2_ alone without undertaking additional and separate sub-ambient O_2_ and elevated CO_2_ growth experiments using the same species. For the purposes of this analysis, suffice it to say that any SD and *D*_v_ responses are the result of the specific O_2_:CO_2_ ratio in the palaeo-treatment growth chambers.

**FIGURE 5:**
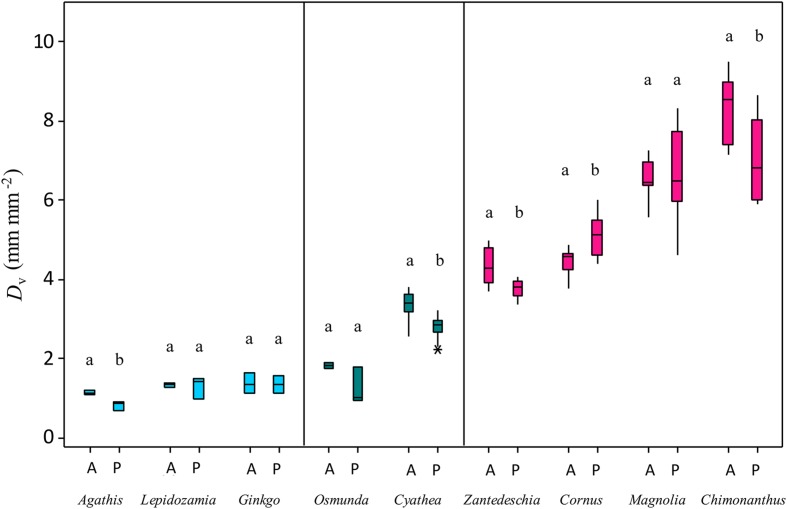
**Comparison of the *D*_v_ responses of gymnosperms, ferns, and angiosperms to growth in low O_2_/high CO_2_ conditions**. Different letters signify a significant difference between treatments. A, ambient treatment; P, palaeo-treatment. ^∗^symbols denote outliers.

It is noteworthy that only two of the studied species (*Chimonanthus praecox* and *Magnolia delavayi*) have vein densities higher than 6 mm mm^-2^ (**Figure 2B**), the ‘critical vein density’ (Feild et al., 2011a; de Boer et al., 2012) that angiosperms are thought to have surpassed as they rose to dominance in the Cretaceous. This emphasizes the similarity between our chosen study species and those very early evolving angiosperms that had vein densities as low as non-angiosperms (Brodribb and Feild, 2010; Boyce and Zwieniecki, 2012). The significant *D*_v_ decrease seen in the fern species (*Cyathea australis*) is interesting (**Figure 2B**), as it is thought that non-angiosperm species are incapable of altering their vein architecture in the same way that angiosperm species can (Boyce et al., 2009; de Boer et al., 2012). Non-angiosperms seem to exhibit limited plasticity in *D*_v_ when exposed to a long-term palaeo-atmospheric treatment (**Figure 5**). Examination of archived leaf material of three gymnosperms and one fern species from a 2009 palaeo-experiment (Haworth et al., 2013) shows that growth in a low O_2_/high CO_2_ atmosphere results in a change in *D*_v_ in one gymnosperm species (*Agathis australis*), but has no effect on *D*_v_ in the remaining species (two gymnosperms and one fern). These non-angiosperms have vein densities below 2 mm mm^-2^ and all have either parallel or dichotomously branching major vein networks, implying that this vein configuration may lack developmental plasticity. The major veins of non-angiosperms are generally thicker in diameter and their xylem anatomy is distinct from that of the angiosperms, lacking an important feature that is believed to be paramount in the proliferation of minor veins, vessels with simple perforation plates. Only angiosperms evolved these less resistive perforation plates, and this in conjunction with the development of thinner minor veins may have allowed this plant group to outperform non-angiosperms (Feild and Brodribb, 2013). Angiosperms also possess vein endings that are diffuse or dispersed throughout the leaf allowing them to develop more reticulate venation patterns, whereas gymnosperms with their marginal vein endings lack this ability (Boyce, 2005). An important implication of the current findings is that some angiosperm species are able to alter their vein density on a developmental time-scale in response to a change in atmospheric composition; studies to date have only discussed CO_2_-driven *D*_v_ changes across evolutionary time-scales (Brodribb and Feild, 2010; Boyce and Zwieniecki, 2012; McElwain et al., 2016b). Results of the current study indicate that at least in some early diverging species, *D*_v_ is a plant functional trait that can respond dynamically to atmospheric change.

### Relationship between *D*_v_, SD, and *g*_max_

The strong relationship observed in both the ambient and palaeo-treatment between *D*_v_ and SD (**Figure 4A**) demonstrates inter-specific co-ordination in two morphological plant traits that determine hydraulic supply and demand across different plant lineages and under a changing atmosphere. Furthermore, the robust relationship observed between *D*_v_ and *g*_max_ (**Figure 4B**) demonstrates that morphology has the potential to influence the physiological behavior of these species, via the strong relationship already found between *g*_max_ and *g*_op_ (Franks et al., 2009; Dow et al., 2014; McElwain et al., 2016b). Examining the direction of change in the morphological traits for each species it is clear that a high degree of intra-specific co-ordination is also occurring (**Figure 3**). Three out of four angiosperm species and the fern species show intra-specific co-ordination in *D*_v_, SD, and *g*_max_ in response to the palaeo-treatment. Co-ordination between traits that determine the water relations (supply and demand) of a plant is critical for its survival. For example, an increase in SD and/or *g*_max_ would increase the evaporative demands of the plant and without a corresponding increase in *D*_v_ (to match the increase in water demand with an increase in hydraulic supply) the plant would be mal-adapted to its environment and would most likely not survive. The opposite scenario would not be as detrimental to plant survival, however, a decrease in SD and/or *g*_max_ without a corresponding decrease in *D*_v_ would result in a waste of resources, the construction of veins being costly to the plant (Sack and Scoffoni, 2013). This ability to co-ordinate morphological traits under a changing atmosphere likely occurred throughout plant evolutionary history as the composition of atmospheric O_2_ and CO_2_ fluctuated, allowing certain plant species to adapt and survive. During the Cretaceous decline in atmospheric CO_2_ for example, it is thought that angiosperms were able to increase their gas exchange capacity (thereby increasing photosynthetic rates) by evolving smaller stomata (Franks and Beerling, 2009a), and by increasing both the density of stomata on the leaf surface and the density of veins (Boyce et al., 2009; Brodribb and Feild, 2010; de Boer et al., 2012; McElwain et al., 2016b). Furthermore, angiosperms that surpassed the ‘critical vein density’ of 6 mm mm^-2^ were able to out-compete the gymnosperms and ferns in niches with high evapotranspirational demand where an increase in water supply to the leaf would have been necessary for survival (de Boer et al., 2012). Higher vein densities have been suggested to confer a higher capacity for CO_2_ uptake and an increased range of *g*_op_; this would explain the ability of angiosperms to expand to such diverse habitats and to outcompete species that are more constrained in their venation and hence gas exchange capacity (McElwain et al., 2016b). A recent study suggests that angiosperms are indeed hydraulically optimized for a diverse range of environments, achieving this by maintaining an equal vein to vein and vein to evaporative surface distance in the leaf (Zwieniecki and Boyce, 2014). Ferns are under-invested hydraulically due to their thin leaves and large vein to vein distances, and while some gymnosperms do approach optimal investment by producing thicker leaves in more water-demanding environments, they are as a group sub-optimal in terms of vein placement (Zwieniecki and Boyce, 2014).

The current study supports these theories by demonstrating a higher degree of plasticity in *D*_v_ in some early diverging angiosperms in response to a changing O_2_:CO_2_ ratio, compared to the studied gymnosperms and ferns (**Figure 5**). It is interesting, however, that examined non-angiosperm species from the 2009 palaeo-experiment show limited plasticity in *D*_v_ as well as in SD (see SD results for these non-angiosperm species reported in Haworth et al., 2013). This demonstrates that while these species do not show a high degree of morphological plasticity in response to a changing atmosphere on experimental time-scales comparable to the studied angiosperms, they do demonstrate similar co-ordination in leaf morphological traits.

Furthermore, these results suggest that angiosperms are not only capable of showing morphological plasticity in response to rising O_2_ and declining CO_2_ (Boyce et al., 2009; Feild et al., 2011a; de Boer et al., 2012), but also to declining O_2_ and high CO_2_ conditions. The robust positive relationship observed here between the density of veins and stomata strongly supports the theory suggested by Brodribb et al. (2007), whereby multi-veined leaves optimize the placement of veins in relation to stomata so that the distance water needs to travel through the resistive mesophyll (*D*_m_) is minimized. Although *D*_m_ was not directly measured in this study, the morphological co-ordination observed suggests that any change in *D*_v_ will elicit a corresponding change in SD or vice versa, allowing the leaf to minimize the distance between veins and stomata, and to maximize photosynthetic performance and operational efficiency of the leaf.

### Implications for Past Plant–Atmosphere Interactions

The experimental species examined show a species-specific and varied response to growth in the palaeo-treatment, yet the strong positive relationship between *D*_v_ and SD persists (**Figure 4A**). Furthermore, the positive relationship observed between *D*_v_ and *g*_max_ (**Figure 4B**) demonstrates the link between hydraulic and gas exchange/diffusional processes in these species, as shown in previous studies (Sack et al., 2003; Boyce et al., 2009; Brodribb and Feild, 2010; Feild et al., 2011b; McElwain et al., 2016b). The finding that *D*_m_ is most likely maintained under changing atmospheric conditions (due to the intra-specific co-ordination between *D*_v_ and SD) has important implications when attempting to understand plant–atmosphere interactions throughout the last 400 million years of plant evolution. A lack of co-ordination in *D*_v_ and SD on developmental time-scales would result in a plant that is morphologically and physiologically out of sync, negatively impacting operational efficiency and overall fitness under changing atmospheric conditions. Furthermore, plant species that exhibited plasticity in these morphological traits under a changing atmosphere would likely have had an ecological advantage over plant species that were morphologically inflexible, being able to maximize their photosynthetic capacity as the surrounding environment changed (McElwain et al., 2016b). The finding that a proxy for photosynthetic capacity (*D*_m_) (Brodribb et al., 2007) remains stable under changing atmospheric conditions is important for accurate initial parameterisation of mechanistically based models used to predict palaeo-CO_2_ (Franks et al., 2014) since these require robust estimates of palaeo-assimilation (Franks et al., 2014; McElwain et al., 2016a). The current study focuses on plant responses to an experimentally imposed low O_2_/high CO_2_ atmosphere. This O_2_:CO_2_ ratio occurred multiple times throughout plant evolutionary history based on model and proxy estimates (Royer, 2001; Bergman et al., 2004; Royer et al., 2004; McElwain et al., 2005; Berner, 2006, 2009; Steinthorsdottir et al., 2016), however, it would be beneficial to investigate the effect of other atmospheric O_2_:CO_2_ ratios on these plant trait relationships in order to test their linearity. Further experiments examining plant responses to a range of palaeo-atmospheric conditions will build on these results, providing a better picture of plant-atmosphere interactions over the past 400 million years and allowing predictions of future plant responses to global climate change.

## Conclusion

Species show a varied response in SD, *D*_v_, and *g*_max_ to growth in an experimental low O_2_/high CO_2_ palaeo-atmosphere. Regardless of this variation in responses, a strong relationship is observed between *D*_v_ and SD and *D*_v_ and *g*_max_ under both the ambient and palaeo-atmosphere. Gymnosperms studied here appear to lack the same degree of developmental plasticity in *D*_v_ compared to the angiosperms, at least on short experimental time-scales. The ability to increase their range of *D*_v_ values may have contributed to the success of angiosperms during the Cretaceous decline in CO_2_; a high degree of plasticity in this trait possibly provided early diverging angiosperms with a competitive advantage over other seed plant groups in more changeable environments. The tight relationship observed between *D*_v_ and SD in the palaeo-treatment suggests that *D*_m_ is likely maintained under environmental change and lends confidence to existing palaeo-CO_2_ proxies that use this parameter in their models. Further studies examining the robustness of these plant trait relationships under a range of O_2_:CO_2_ ratios are needed in order to elucidate the full spectrum of plant–atmosphere interactions throughout the last 400 million years.

## Author Contributions

CE-F carried out vein density and SD analysis, anatomical stomatal measurements for *g*_max_ calculation, statistical analysis, and drafted the manuscript. AP contributed SD and *g*_max_ data for *Cyathea australis*. CE-K was involved in the 2009 palaeo-experiment and therefore provided dried leaf material for vein density analysis. JM is the principal investigator. All authors read, revised, and approved the final manuscript.

## Conflict of Interest Statement

The authors declare that the research was conducted in the absence of any commercial or financial relationships that could be construed as a potential conflict of interest.
