# Supplementary material for: Co-ordination in Morphological Leaf Traits of Early Diverging Angiosperms Is Maintained Following Exposure to Experimental Palaeo-atmospheric Conditions of Sub-ambient O2 and Elevated CO2
Source: Front Plant Sci. 2016 Sep 15;7:1368. doi: 10.3389/fpls.2016.01368 (PMC5023689; doi:10.3389/fpls.2016.01368)
Supplement: Supplementary file 1 [file Table_1.DOCX]

**Table S1.** Classification, pot size, substrate type, and fertiliser used for experimental species. Osmocote (Scotts, Marysville, OH, USA) composition is 15 % N, 10 % P_2_O_5_, 10 % K_2_O, 2 % MgO, plus trace elements.

|  | Fern | Angiosperms | | | |
| --- | --- | --- | --- | --- | --- |
| Order | Cyatheales | Magnoliales | Laurales | Alismatales | Cornales |
| Family | Cyatheaceae | Magnoliaceae | Calycanthaceae | Araceae | Cornaceae |
| Genus | Cyathea | Magnolia | Chimonanthus | Zantedeschia | Cornus |
| Species | *C.australis* | *M.delavayi* | *C.praecox* | *Z.aethiopica* | *C.capitata* |
| Pot size (Litres) | 3 | 3 | 4 | 4 | 2 |
| Compost mix | **90:10**  Shamrock  multi-purpose  compost:  vermiculite | **90:10**  Shamrock multi-purpose compost: vermiculite | **58:25:17**  Loam: Peat: Sand | **90:10**  Shamrock multi-purpose compost: vermiculite | **90:10**  Shamrock multi-purpose compost: vermiculite |
| Fertiliser | 2 Osmocote plugs  (5-6 months) | 10g/L Osmocote  (12-14 months) | 10g/L Osmocote  (12-14 months) | 10g/L Osmocote  (16-18 months) | 10g/L Osmocote  (12-14 months) |
