# Supplementary material for: Co-ordination in Morphological Leaf Traits of Early Diverging Angiosperms Is Maintained Following Exposure to Experimental Palaeo-atmospheric Conditions of Sub-ambient O2 and Elevated CO2
Source: Front Plant Sci. 2016 Sep 15;7:1368. doi: 10.3389/fpls.2016.01368 (PMC5023689; doi:10.3389/fpls.2016.01368)
Supplement: Supplementary file 2 [file Table_2.DOCX]

**Table S2.** Results of generalised linear models investigating differences in SD, *D*_v_, and *g*_max_ between treatments for each species. F values, degrees of freedom (df) and associated p-values are shown.

|  | *Chimonanthus praecox* | | | *Magnolia delavayi* | | | *Cornus capitata* | | | *Zantedeschia aethiopica* | | | *Cyathea australis* | | |
| --- | --- | --- | --- | --- | --- | --- | --- | --- | --- | --- | --- | --- | --- | --- | --- |
|  | **SD** | ***D*_v_** | ***g*_max_** | **SD** | ***D*_v_** | ***g*_max_** | **SD** | ***D*_v_** | ***g*_max_** | **SD** | ***D*_v_** | ***g*_max_** | **SD** | ***D*_v_** | ***g*_max_** |
| F value | 6.78 | 13.70 | 6.70 | 1.38 | 0.14 | 1.97 | 2.33 | 19.78 | 3.47 | 26.64 | 9.6 | 77.98 | 2.52 | 18.01 | 0.03 |
| df | 1,11 | 1,11 | 1,11 | 1,11 | 1,11 | 1,11 | 1,11 | 1,11 | 1,11 | 1,8 | 1,8 | 1,8 | 1,11 | 1,11 | 1,11 |
| p-value | 0.03 | 0.003 | 0.03 | 0.27 | 0.72 | 0.19 | 0.16 | 0.001 | 0.09 | 0.001 | 0.02 | 0.000 | 0.14 | 0.001 | 0.87 |
